# Four new spider species of the family Theridiosomatidae (Arachnida, Araneae) from caves in Laos

**DOI:** 10.3897/zookeys.391.7161

**Published:** 2014-03-20

**Authors:** Yucheng Lin, Shuqiang Li, Peter Jäger

**Affiliations:** 1 Key Laboratory of Bio-resources and Eco-environment (Ministry of Education), College of Life Sciences, Sichuan University, Chengdu, Sichuan 610064, China; 2 Institute of Zoology, Chinese Academy of Sciences, Beijing 100101, China; 3 Arachnology, Senckenberg Research Institute, Senckenberganlage 25, 60325 Frankfurt am Main, Germany

**Keywords:** Cave spiders, taxonomy, Araneoidea, Orbiculariae, Indochina, limestone karst

## Abstract

Four new species of the spider family Theridiosomatidae are described from caves in Laos: *Alaria cavernicola* **sp. n.** (♂♀), *A. navicularis* **sp. n.**, (♂♀) *A. bicornis* **sp. n.** (♂♀), *Chthonopes thakekensis* **sp. n.** (♀). Diagnoses and illustrations for all new taxa are given. All holotypes are deposited in the Senckenberg Research Institute in Frankfurt am Main, Germany (SMF).

## Introduction

Theridiosomatidae are small (usually ≤ 3 mm), widely distributed, and cryptozoic spiders, which can be found in damp, dark habitats (litter layer of forest or in caves) (Zhao and Li 2012). Coddington (1986) reviewed the genera of this family and proposed synapomorphies based on a cladistic analysis, including the combination of following characters: a pair of pits on the anterior margin of sternum near the labial base (absent in *Chthonos* Coddington, 1986), partly fused spermathecae (separated in *Coddingtonia* Miller, Griswold & Yin, 2009), especially large male palps (except for *Menglunia* Zhao & Li, 2012), and a long trichobothrium dorsally on tibia IV. Subsequently, an increasing number of new species have been described from all over the world. For example, some species from Latin America have been reported by Lopez (1994, 1996), Rodrigues and Ott (2005) and Rodrigues and Lise (2008). Chinese Theridiosomatidae have been reported from Gaoligongshan, Yunnan (Miller et al. 2009), tropical rainforest in Hainan and Xishuangbanna of Yunnan, and in caves in Guangxi, Chongqing and Guizhou (Zhao and Li 2012; Dou and Lin 2012; Chen 2010; Zhu and Wang 1992; Song and Zhu 1994). Other species have been described from insular states or areas (Barrion and Litsinger 1995; Saaristo 1996; Zhang et al. 2006; Shinkai 2009). According to the latest data, 18 genera containing 101 known species are recorded worldwide (Platnick 2014).

The earliest report on Theridiosomatidae from the Indochinese Peninsula was published by Simon (1901), who described two species, *Andasta cyclosina* and *Theridiosoma nebulosum* from Malaysia. About one hundred years later, two new genera (*Chthonopes* and *Luangnam*) were established by Wunderlich (2011) to accommodate three new species (*Chthonopes cavernicolus*, *Chthonopes jaegeri* and *Luangnam discobulbus*) discovered from caves in Laos. Insufficient sampling could not hide the rich species diversity of this region, and still more species are waiting to be found. In this paper, we provide detailed descriptions, illustrations and distribution data for four new species from Laos.

## Material and Methods

Specimens were examined and measured under a Leica M205 C stereomicroscope. Further details were studied under an Olympus BX43 compound microscope. All drawings were made using a drawing tube attached to an Olympus BX43 compound microscope, and then inked on ink jet plotter paper. Copulatory organs of males and females were examined and illustrated after they have been dissected and detached from the spiders’ bodies. Vulvae were treated in lactic acid before illustration. All embolic divisions and vulvae were illustrated after being embedded in Hoyer’s Solution. Photos were taken with a Canon EOS 60D wide zoom digital camera (8.5 megapixels) mounted on an Olympus BX43 stereomicroscope. The images were montaged using Helicon Focus 3.10 software. All type specimens are preserved in 85% ethanol solution. All material was collected by Peter Jäger by hand. Material is deposited in Senckenberg Research Institute, Frankfurt, Germany (SMF) and in the Institute of Zoology, Chinese Academy of Sciences, Beijing, China (IZCAS).

All measurements were made in millimeters; altitude is given in meters (m). Leg measurements are given as: total length (femur, patella, tibia, metatarsus, tarsus). The terminology mostly follows Miller et al. (2009) and Zhao and Li (2012). Chaetotaxy of macrosetae is marked for dorsal (d), prolateral (p), retrolateral (r), and ventral (v) surfaces of legs. Metatarsal trichobothrium (Tm) is given as the ratio of the distance between the proximal margin of the metatarsus and the base of the trichobothrium divided by the total length of the metatarsus (Locket and Millidge 1953) and Tm value for each leg is given as Tm I, Tm II, Tm III, and Tm IV. The course of the duct system is illustrated as red line with a circle representing the copulatory opening and an arrow representing the fertilization duct pointing in direction of the Uterus externus.

Abbreviations used in the text: AME – anterior median eyes; DS – dorsal shield of prosoma; LE – lateral eyes; PME – posterior median eyes.

## Taxonomy

### Genus Alaria

Zhao & Li, 2012

http://species-id.net/wiki/Alaria\according_to_Lin_et_al_2014

#### Type species.

*Alaria chengguanensis* Zhao & Li, 2012 from China.

The genus was described in 2012 as monotypic (Zhao and Li 2012). The type species was known from Guizhou Province only from the type locality. Spiders were recorded in Xiniu Cave.

### Alaria cavernicola sp. n.

http://zoobank.org/87575CFC-446E-4846-9D28-E483E9DBC2A0

http://species-id.net/wiki/Alaria_cavernicola

**Figure 1.**
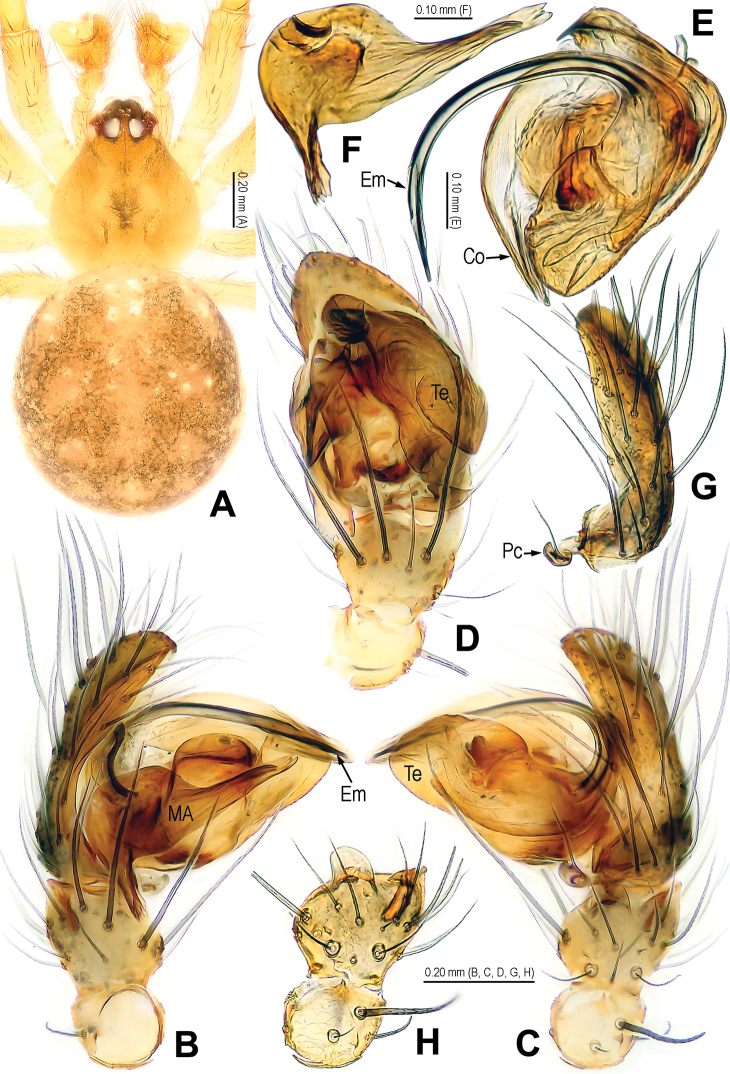
*Alaria cavernicola* sp. n., holotype male. **A** Habitus, dorsal **B** Palp, prolateral **C** Ditto, retrolateral **D** Ditto, ventral **E** bulb (median apophysis removed), distal **F** Median apophysis, prolateral **G** Cymbium, retrolateral **H** Palpal patella and tibia, retrolateral. Co = conductor; Em = embolus; MA = median apophysis; Pc = paracymbium; Te = tegulum.

Figs 1 [Fig F2] [Fig F3] –4 , 19

#### Material examined.

**Laos: *Bolikhamsay Province*:** Holotype: ♂ (SMF), Lak Sao, Tham Man Kone, 18°13.268'N, 104°48.765'E, altitude 501 m, inside cave, leg. 3 December 2012. Paratypes: 1 ♂, 2 ♀ (SMF), same data as holotype; ***Khammouan Province*:** 6 ♂ (SMF), 8.3 km NE of Thakek, Tham Noi, 17°26.655'N, 104°51.767'E, altitude 158 m, in foot cave, leg. 26 November 2012; 1 ♂ (IZCAS), 15 km N of Thakek, Ban Phôungam-Mai,17°31.835'N, 104°46.582'E, altitude 144 m, limestone cave, quarry, leg. 25 November 2012; 1 ♂ (SMF), LAOS, 2.5 km WNW of Ban Tathot, entrance 17°37.897'N, 105°07.502'E, exit 17°37.994'N, 105°07.195'E, altitude 200 m, entrance area and in front of limestone cave, Tham Kamouk, leg. 30 April 2012; 2 ♀ (SMF, IZCAS), Thakek area, Ban Phôungam-Mai, 17°32.954'N, 104°48.754'E, altitude 180 m, limestone cave, Tham Phayat, leg. 29 April 2012; 1 ♀ (SMF), Boualapha District, Tham Nam, Lot Xe Bang Fai, 17°22'24.43"N, 105°50'39.36"E, altitude 190 m, in day, leg. 3–4 May 2012.

#### Etymology.

The specific epithet is derived from the Latin word “cavernicola” = “living in caves”, refers to that this species may mainly live in caves; adjective.

#### Diagnosis.

**Figure 2. F2:**
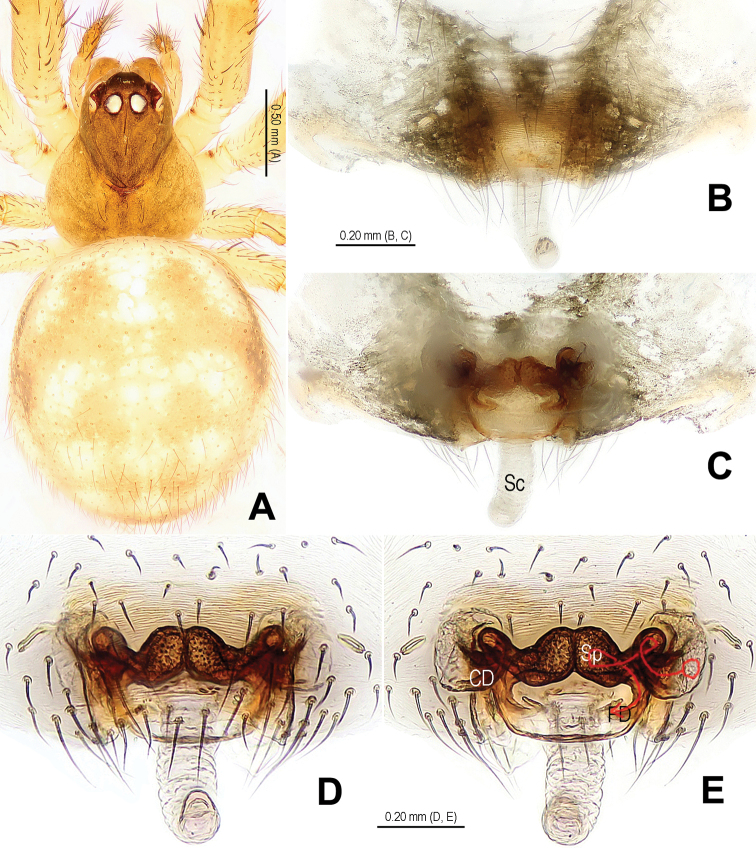
*Alaria cavernicola* sp. n., female paratype, from Tham Man Kone. **A** Habitus, dorsal **B** Epigyne, ventral **C** Epigyne, dorsal **D** Vulva (lactic acid-treated), ventral **E** Vulva, dorsal (red line showing course of duct system). CD = copulatory duct; FD = fertilization duct; Sc = scape; Sp = spermathecae.

**Figure 3. F3:**
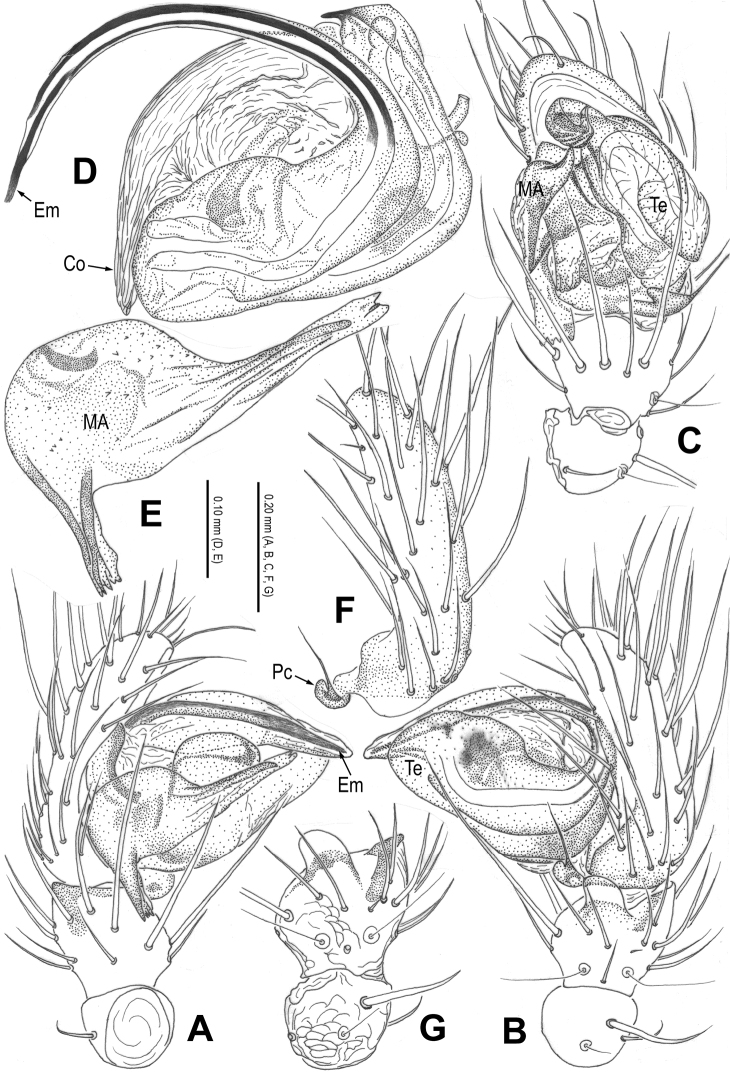
*Alaria cavernicola* sp. n., holotype male. **A** Palp, prolateral **B** Ditto, retrolateral **C** Ditto, ventral **D** bulb (median apophysis removed), apical **E** Median apophysis, prolateral **F** Cymbium, retrolateral **G** Palpal patella and tibia, retrolateral. Co = conductor; Em = embolus; MA = median apophysis; Pc = paracymbium; Te = tegulum.

**Figure 4.**
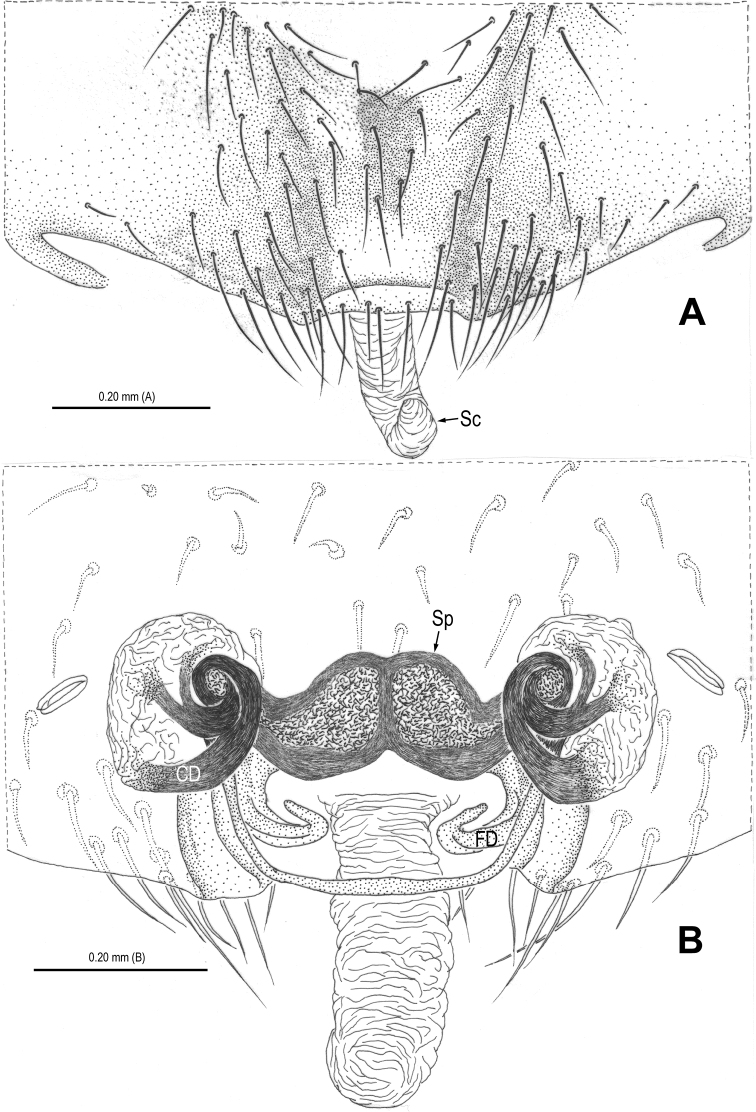
*Alaria cavernicola* sp. n., female paratype, from Tham Man Kone. **A** Epigyne, ventral **B** Vulva (lactic acid-treated), dorsal. CD = copulatory duct; FD = fertilization duct; Sc = scape; Sp = spermathecae.

This new species is similar to *Alaria chengguanensis* Zhao & Li, 2012 in the paracymbial shape (Figs 1D, 3C), most part of the long embolus embedded in conductor, the large median apophysis (Figs 1B–C, 3A–B), the scape protruding from beneath epigynal plate (Figs 2B–E, 4A–B), and the similar configurations of the vulva (Figs 2E, 4B). Males can be distinguished by the absence of tufted setae on the cymbium (Figs 1G, 3F vs. Zhao and Li 2012: figs 1B, 1D, 3D), the different shape of the median apophysis (Figs 1F, 3E vs. Zhao and Li 2012: figs 1A, 3A, 5A), females by the long, narrow and membranous scape as well as by the large and wide spermathecae (Figs 2B–E, 4A–B vs. Zhao and Li 2012: figs 2A–B, 5C–D).

#### Description.

Habitus as in Figs 1A, 2A. DS nearly pear-shaped, yellow, with grey pigment, cervical groove distinct, thoracic fovea present. Sternum yellow. Legs proximally pale yellow, distally yellow. Opisthosoma sub-spherical, grey in male, off-white in female, with white patches.

Male palp (Figs 1B–D, 3A–C): patella with strong macroseta and trichobothrium (Figs 1C, 1H, 3B, 3G). Tibia with 2 trichobothria and a lamellar process (Figs 1H, 3G). Paracymbium with a hooked basal process and a long distal spicula (Figs 1C–D, 1G, 3B–C, 3F). Tegulum smooth. Median apophysis large, surface with tiny grooves and aculei, proximal process short and serrated, distal process long and slightly furcated (Figs 1F, 3E). Most part of embolus embedded in sheath-like conductor (Figs 1B–D, 3A–C). Embolus long, bent, filiform (Figs 1E, 3D).

Female copulatory organ (Figs 2B–E, 4A–B): epigynal area with long setae. Scape long, membranous, rugose, distally bent, protruding from beneath epigynal posterior margin (Figs 2D, 4A). Spermathecae subovate, juxtaposed (Figs 2C, 2E, 4B). Copulatory ducts wide, starting from lateral corner of spermathecae (Fig. 4B), curl up to form a saccular structure at each side (Figs 2E, 4B). Fertilization ducts deriving from ventral surface of spermathecae, distally hooked (Fig. 4B).

Male: total length 1.52, DS 0.73 long, 0.60 wide, clypeus 0.16, sternum 0.39 long, 0.33 wide, coxae IV separated by their width, opisthosoma 0.82 long, 0.75 wide. PME separated by less than half their diameter. Macrosetae: leg I: femur d1, p1, r1, patella d2, tibia d2, p2, r1, v1, metatarsus d1, p1, r1; leg II: femur d1, r1, patella d2, tibia d2, p1, r1, metatarsus d1, r1; leg III: femur d1, patella d2, tibia r1, metatarsus d1, p1, r1; leg IV: femur d2, patella d2, tibia d2, p1, metatarsus d1, p1, r1. Metatarsal trichobothria: Tm I: 0.29; Tm II: 0.25; Tm III: 0.14. Leg measurements: I 2.02 (0.65, 0.28, 0.40, 0.41, 0.28); II 1.64 (0.51, 0.25, 0.32, 0.32, 0.24); III 1.14 (0.34, 0.19, 0.18, 0.23, 0.20); IV 1.48 (0.45, 0.22, 0.30, 0.29, 0.22).

Female (collected together with holotype, from Tham Man Kone): total length 2.65, DS 1.03 long, 0.99 wide, clypeus 0.18, sternum 0.62 long, 0.53 wide, coxae IV separated by their width, opisthosoma 1.67 long, 1.58 wide. PME separated by less than half their diameter. Macrosetae: leg I: femur d1, p1, r1, patella d2, tibia d2, p2, r1, v2, metatarsus p1, r1, v1; leg II: femur d1, r1, patella d2, tibia d2, p1, r1, v1, metatarsus p1, r1, v1; leg III: femur d1, patella d2, tibia d1, r1, metatarsus d1, p1, r1, v1; leg IV: femur d2, patella d2, tibia d2, p1, r1, v1, metatarsus p1, r1. Metatarsal trichobothria: Tm I: 0.27; Tm II: 0.27; Tm III: 0.16. Leg measurements: I 3.48 (1.18, 0.47, 0.71, 0.71, 0.41); II 2.92 (0.93, 0.42,0.58, 0.60, 0.39); III 1.99 (0.59, 0.31, 0.34, 0.43, 0.32); IV 2.79 (0.93, 0.38, 0.56, 0.55, 0.37).

#### Variation.

The total length ranges from 1.48 to 1.62 in males (n = 10) and from 2.38 to 2.70 in females (n = 5).

#### Distribution.

See in Fig. 19.

### Alaria navicularis sp. n.

http://zoobank.org/05825720-58CD-40F4-9FBE-4FBA4A97E5D1

http://species-id.net/wiki/Alaria_navicularis

**Figure 5.**
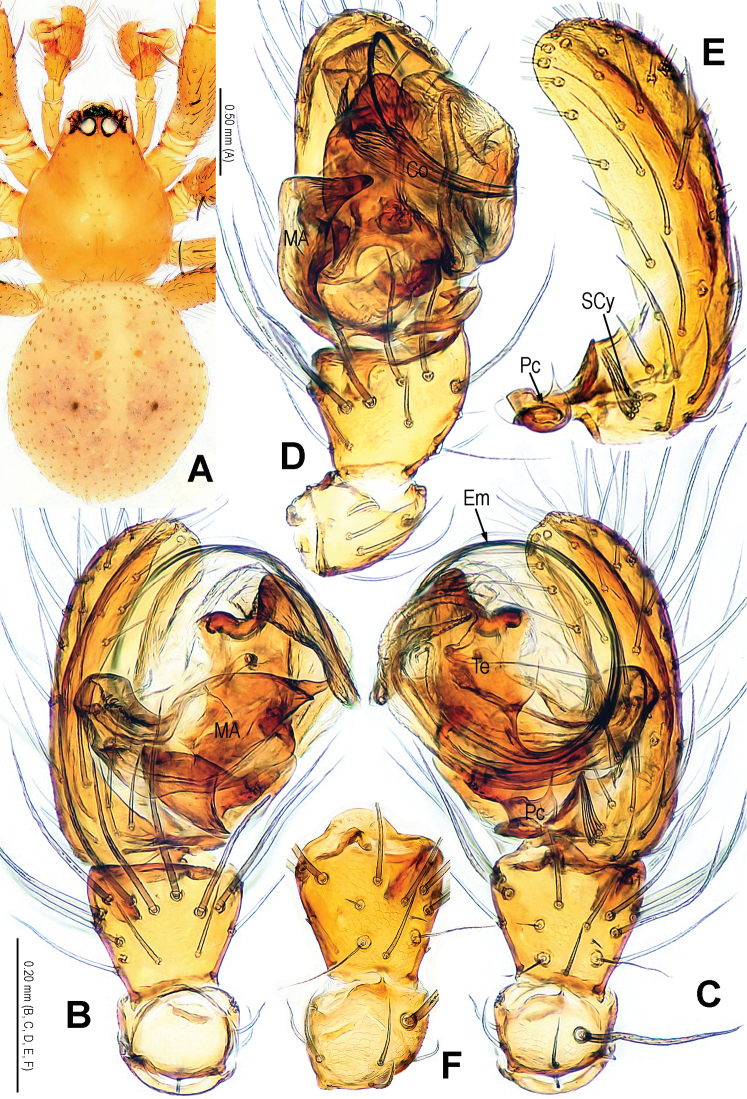
*Alaria navicularis* sp. n., holotype male. **A** Habitus, dorsal **B** Palp, prolateral **C** Ditto, retrolateral **D** Ditto, ventral **E** Cymbium, retrolateral **F** Palpal patella and tibia, retrolateral. Co = conductor; Em = embolus; MA = median apophysis; Pc = paracymbium; SCy = setae of cymbium; Te = tegulum.

Figs 5 [Fig F6] [Fig F7] [Fig F8] [Fig F9] –10 , 19

#### Material examined.

**Laos: *Khammouan Province*:** Holotype: ♂ (SMF), 2.5 km WNW of Ban Tathot, entrance 17°37.897'N, 105°07.502'E, exit 17°37.994'N, 105°07.195'E, altitude 200 m, entrance area and in front of limestone cave, Tham Kamouk, leg. 30 April 2012. Paratypes: 2 ♀ (SMF), same data as holotype; 1 ♀ (SMF), 8.3 km NE of Thakek, Tham Noi, 17°26.655'N, 104°51.767'E, altitude 158 m, in foot cave, leg. 26 November 2012; 1 ♀ (IZCAS), 2.5 km WNW of Ban Tathot, entrance 17°37.897'N, 105°07.502'E, exit 17°37.994'N, 105°07.195'E, altitude 200 m, entrance area and inner parts of limestone cave, Tham Kamouk, leg. 26 April 2012.

#### Etymology.

This specific name is derived from the Latin word “navicularis” = “shaped like a boat”, and refers to the shape of the median apophysis in the male palp; adjective.

#### Diagnosis.

The most significant difference of this new species to *Alaria chengguanensis* (Zhao and Li 2012: figs 1A–D, 2A–B, 3A–D, 4A–B, 5A–B) and *Alaria cavernicola* sp. n. (Figs 1–4) is the navicular median apophysis in the male (Figs 5B, 6D, 8A, 9D), the triangular, weakly sclerotized scape and the nearly circular, juxtaposed spermathecae in the female (Figs 7B–D, 10A–C).

**Figure 6. F6:**
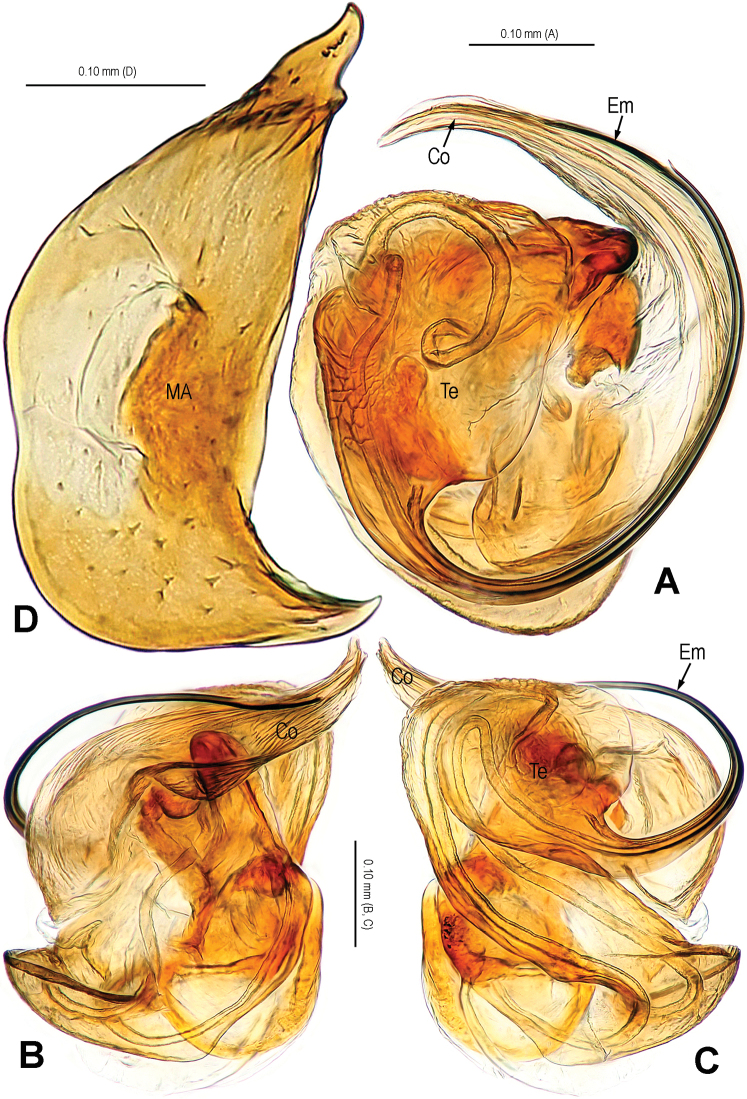
*Alaria navicularis* sp. n., male holotype. **A** Bulb (median apophysis removed), distal **B** Ditto, prolateral **C** Ditto, retrolateral **D** Median apophysis, prolateral. Co = conductor; Em = embolus; MA = median apophysis; Te = tegulum.

**Figure 7. F7:**
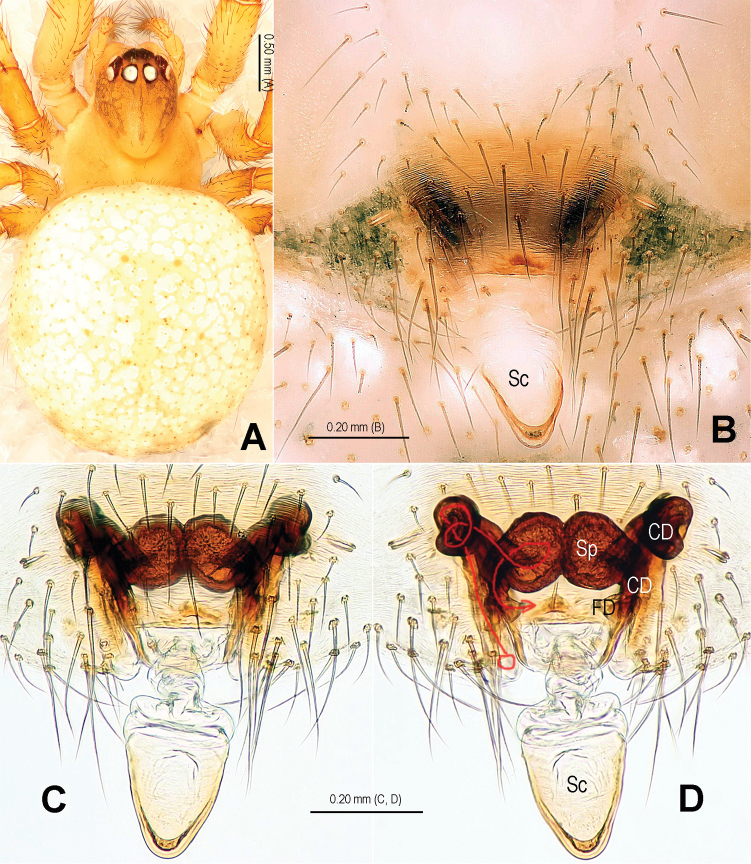
*Alaria navicularis* sp. n., female paratype, from Tham Kamouk. **A** Habitus, dorsal **B** Epigyne, ventral **C** Vulva (lactic acid-treated), ventral **D** Ditto, dorsal (red line showing course of duct system). CD = copulatory duct; FD = fertilization duct; Sc = scape; Sp = spermathecae.

**Figure 8. F8:**
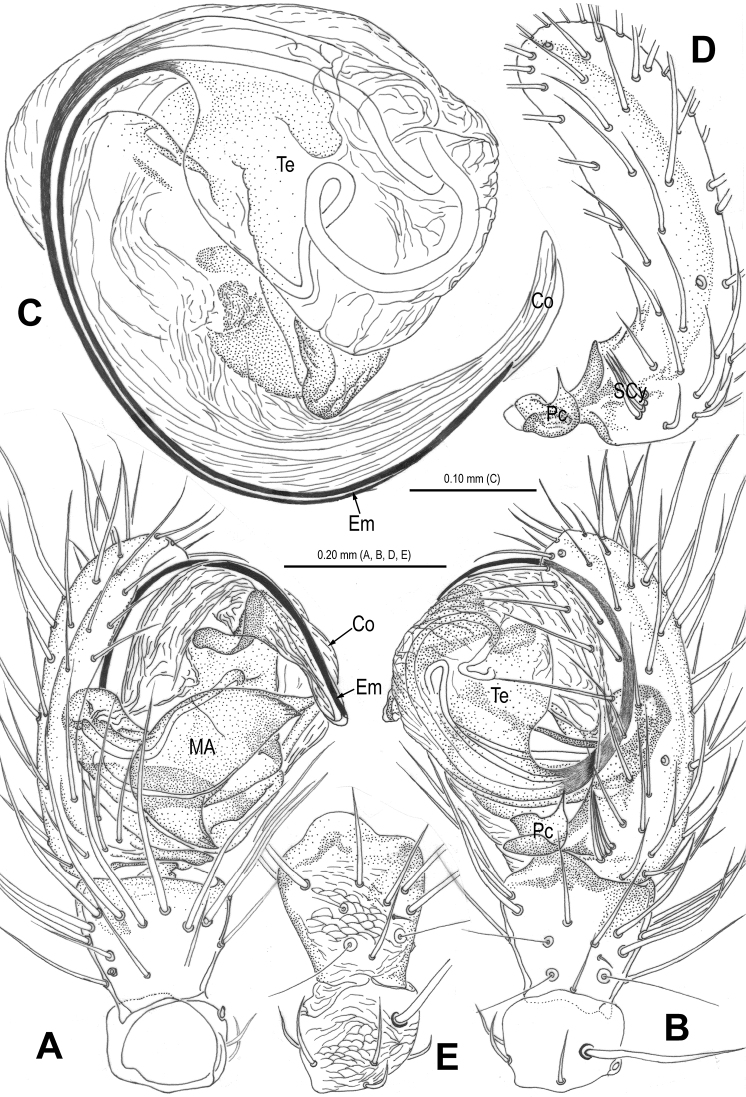
*Alaria navicularis* sp. n., holotype male. **A** Palp, prolateral **B** Ditto, retrolateral **C** Bulb (median apophysis removed), distal **D** Cymbium, retrolateral **E** Palpal patella and tibia, retrolateral. Co = conductor; Em = embolus; MA = median apophysis; Pc = paracymbium; SCy = setae of cymbium; Te = tegulum.

**Figure 9. F9:**
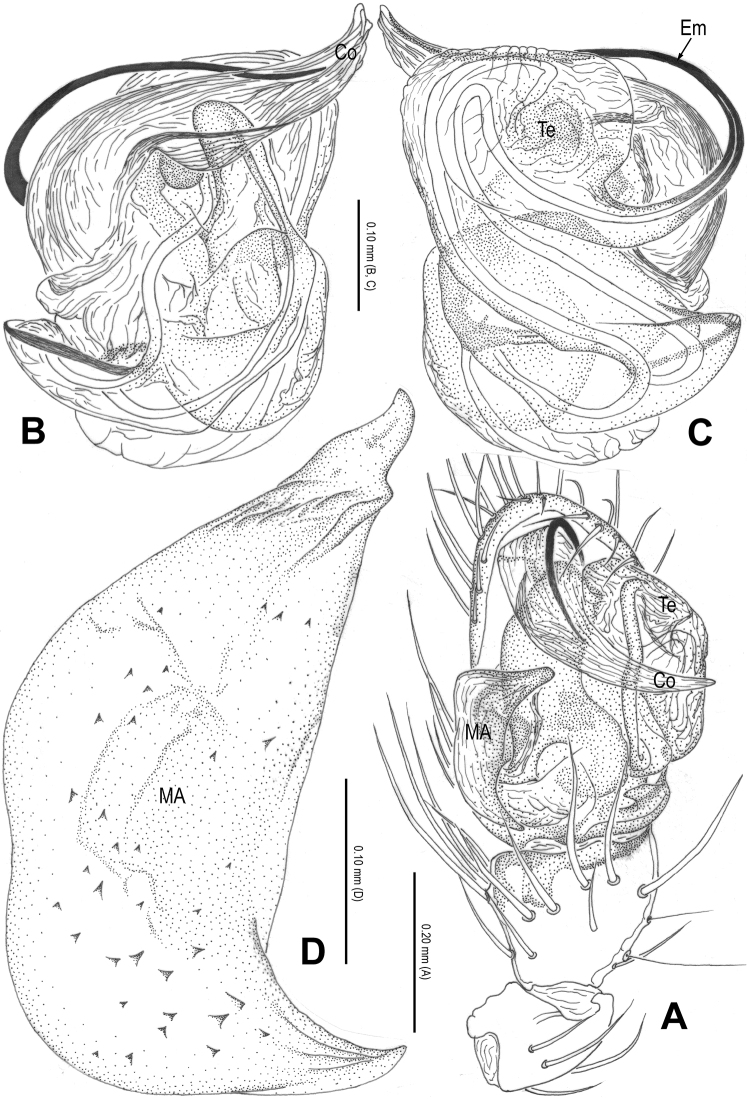
*Alaria navicularis* sp. n., holotype male. **A** Palp, ventral **B** Bulb (median apophysis removed), prolateral **C** Ditto, retrolateral **D** Median apophysis, prolateral. Co = conductor; Em = embolus; MA = median apophysis; Te = tegulum.

**Figure 10.**
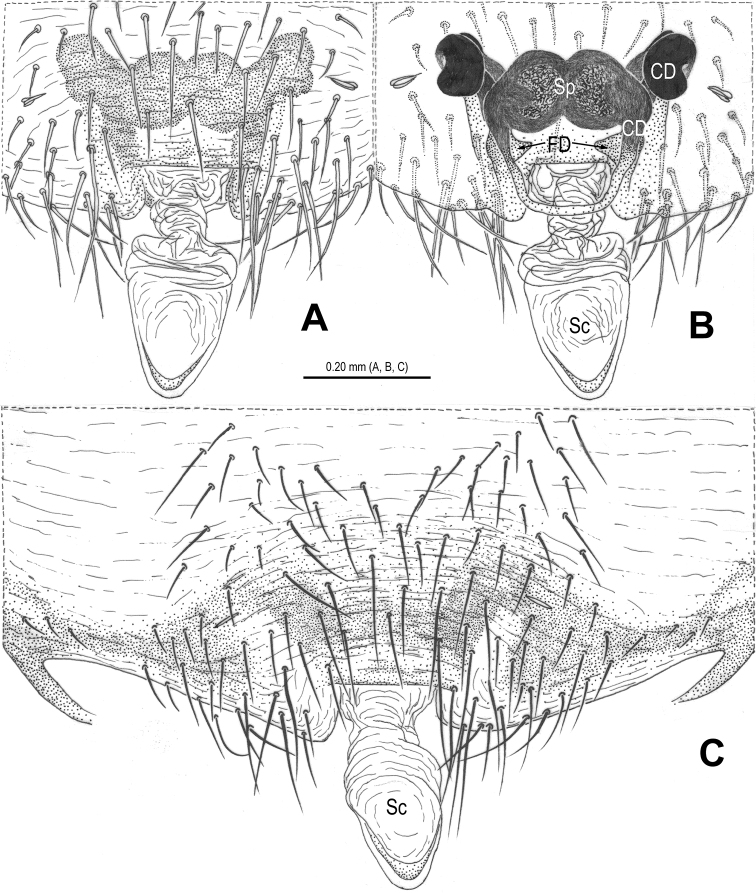
*Alaria navicularis* sp. n., female paratype, from Tham Kamouk. **A** Vulva (lactic acid-treated), ventral **B** Ditto, dorsal **C** Epigyne (untreated), ventral. CD = copulatory duct; FD = fertilization duct; Sc = scape; Sp = spermathecae.

#### Description.

Habitus see in Figs 5A, 7A. DS pear-shaped, yellow, eyes with black base, cervical groove distinct, sternum yellow. Legs yellow. Opisthosoma oval in male, pale yellow, dorsum with small slcerotized spots and a pair of black speckles; sub-spherical in female, pale, dorsum with white patches.

Male palp (Figs 5B–D, 8A–B, 9A): patella with strong macroseta (Figs 5C, 8B, 8E). Tibia with 3 trichobothria and lamellar process (Figs 5F, 8E). Paracymbium with basal hooked process and distal short spicula (Figs 5D–E, 8D, 9A). Tegulum medially smooth, marginally rugose (Figs 6A, 8C). Median apophysis especially large, navicular, surface with tiny aculei, proximal process short, distal process long and rugose (Figs 6D, 9D). Conductor chisel-shaped, distally slightly bent, longer than embolus, (Figs 6B–C, 9B–C). Embolus long, needle-shaped, sclerotized, distal part embedded in conductor, embolic tip not beyond distal end of conductor (Figs 6A–C, 8C, 9A–C).

Female copulatory organ (Figs 7B–D, 10A–C): epigynal area covered with long setae (Figs 7B, 10A, 10C). Scape large, triangular, basally rugose and contracted, apically weakly sclerotized, with small distal pocket, protruding from beneath epigynal posterior margin (Figs 7B–D, 10A–C). Spermathecae sub-circular, juxtaposed; lateral copulatory ducts coils oval, bent (Figs 7D, 10B). Copulatory ducts narrow, ending laterally margin in spermathecae (Figs 7D, 10B). Fertilization ducts short, triangular, arising ventro-laterally from spermathecae (Figs 7D, 10B).

Male: total length 2.31, DS 1.10 long, 0.93 wide, clypeus 0.17, sternum 0.55 long, 0.45 wide, coxae IV separated by their width, opisthosoma 1.31 long, 1.20 wide. PME separated by less than half their diameter. Macrosetae: leg I: femur d2, p1, r1, patella d2, tibia d2, p3, r1, v2, metatarsus d1, p1, r1, v1; leg II: femur d2, r1, patella d2, tibia d2, p1, r1, v1, metatarsus d2, r1, v1; leg III: femur d2, patella d2, tibia d2, p1, v1, metatarsus d1, p1, r1, v1; leg IV: femur d2, p1, patella d2, tibia d2, p1, r1, v1, metatarsus d1, p1, r1, v1. Metatarsal trichobothria: Tm I: 0.22; Tm II: 0.24; Tm III: 0.14. Leg measurements: I 3.34 (1.09, 0.45, 0.67, 0.69, 0.44); II 2.90 (0.93, 0.42, 0.57, 0.58, 0.40); III 2.16 (0.72, 0.32, 0.36, 0.43, 0.33); IV 2.65 (0.85, 0.37, 0.53, 0.55, 0.35).

Female (collected together with holotype): Total length 3.62, DS 1.24 long, 1.45 wide, clypeus 0.15, sternum 0.78 long, 0.68 wide, coxae IV separated by their width, opisthosoma 2.45 long, 2.35 wide. PME separated by less than half their diameter. Macrosetae: Leg I: femur d1, p1, r1, patella d2, tibia d2, p2, r1, v1, metatarsus p1, r1, v1; leg II: femur d2, r1, patella d2, tibia d2, p1, r1, v1, metatarsus p1, r1, v1; leg III: femur d1, v1, patella d2, tibia d2, p1, v1, metatarsus d1, p1, r1, v1; leg IV: femur d2, p1, patella d2, tibia d2, p1, r1, v1, metatarsus d1, p1, r1, v1. Metatarsal trichobothria: Tm I: 0.30; Tm II: 0.25; Tm III: 0.15. Leg measurements: I 4.46 (1.45, 0.61, 0.93, 0.88, 0.59); II 3.85 (1.20, 0.56, 0.78, 0.77, 0.54); III 2.83 (0.92, 0.42, 0.49, 0.54, 0.46); IV 3.54 (1.16, 0.52, 0.70, 0.69, 0.47).

#### Variation.

The total length ranges from 3.42 to 3.70 in females (n = 4).

#### Distribution.

See in Fig. 19.

### Alaria bicornis sp. n.

http://zoobank.org/BA4F961C-149D-4C48-842B-C88A71DE3282

http://species-id.net/wiki/Alaria_bicornis

**Figure 11.**
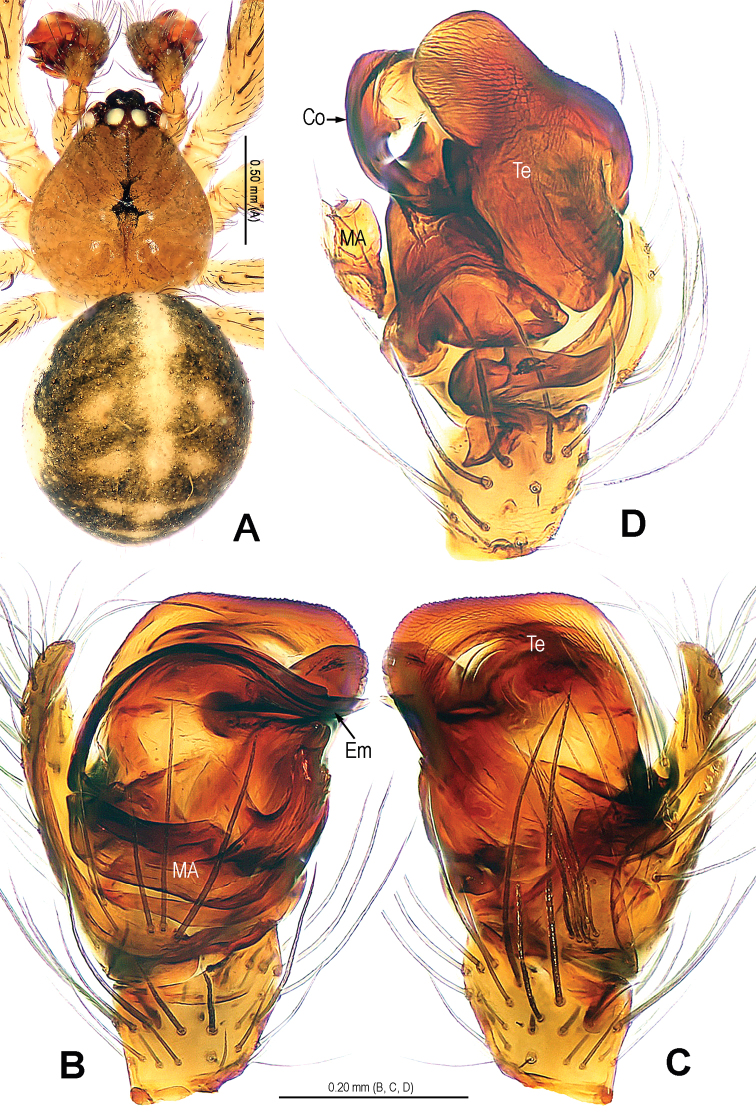
*Alaria bicornis* sp. n., holotype male. **A** Habitus, dorsal **B** Palp, prolateral **C** Ditto, retrolateral **D** Ditto, ventral. Co = conductor; Em = embolus; MA = median apophysis; Te = tegulum.

Figs 11 [Fig F12] [Fig F13] [Fig F14] [Fig F15] –16 , 19

#### Material examined.

**Laos: *Vientiane Province*: Vang Vieng:** Holotype: ♂ (SMF), North of Ban Phoxay, 19°00.880'N, 102°25.902'E, altitude 280 m, Tham Kieo, in cave, leg. 2 December 2012. Paratypes: 2 ♂, 5 ♀ (SMF), with same data as for holotype; 2 ♂, 2 ♀ (SMF, IZCAS), cross Nam Song, 18°54.550'N, 102°26.527'E, altitude 270 m, Tham Xiang, in cave, leg. 3 December 2012; 5 ♀ (SMF), N of Ban Phoxay, 19°02.350'N, 102°25.423'E, altitude 256 m, Tham Hoi, in cave, leg., 3 December 2012; 3 ♀ (SMF), N of Ban Phoxay, 19°01.749'N, 102°25.954'E, altitude 290 m, Tham Phathao, in cave, leg. 3 December 2012.

#### Etymology.

This specific name is derived from the Latin word “bicornis” = “with two horns”, referring to the median apophysis with a fingerlike and a hooked process in the male palp; used as an adjective.

#### Diagnosis.

**Figure 12. F12:**
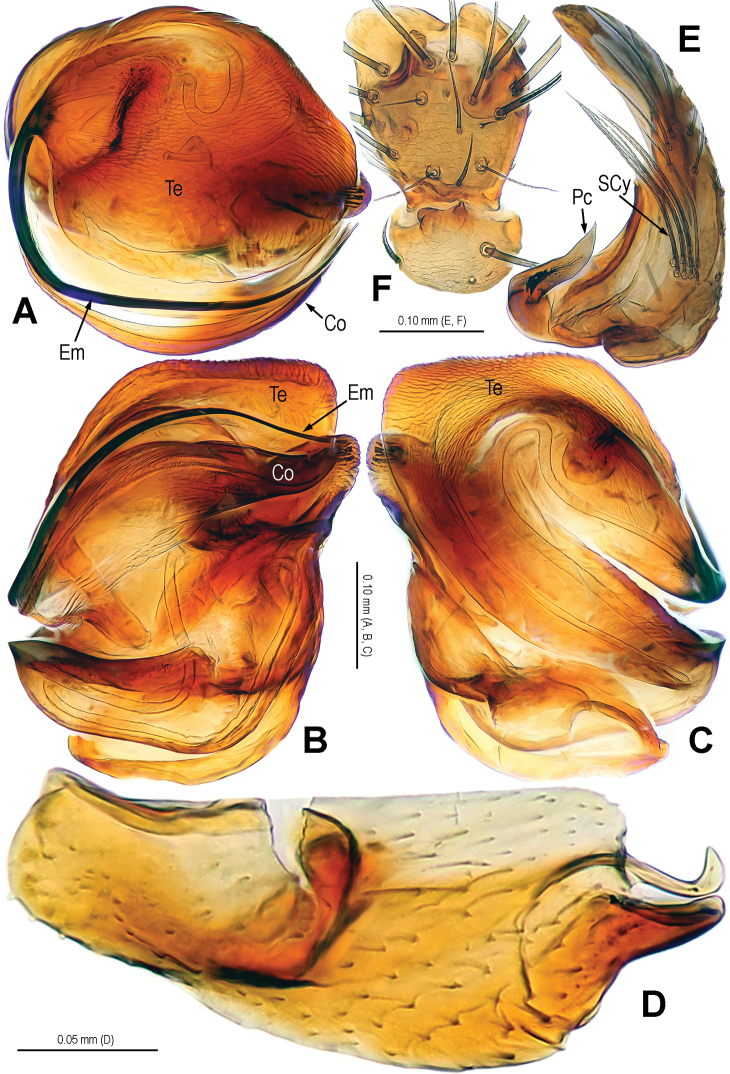
*Alaria bicornis* sp. n., holotype male. **A** Bulb (median apophysis removed), distal **B** Ditto, prolateral **C** Ditto, retrolateral **D** Median apophysis, prolateral **E** Cymbium, retrolateral **F** Palpal patella and tibia, ventral. Co = conductor; Em = embolus; Pc = paracymbium; SCy = setae of cymbium; Te = tegulum.

**Figure 13. F13:**
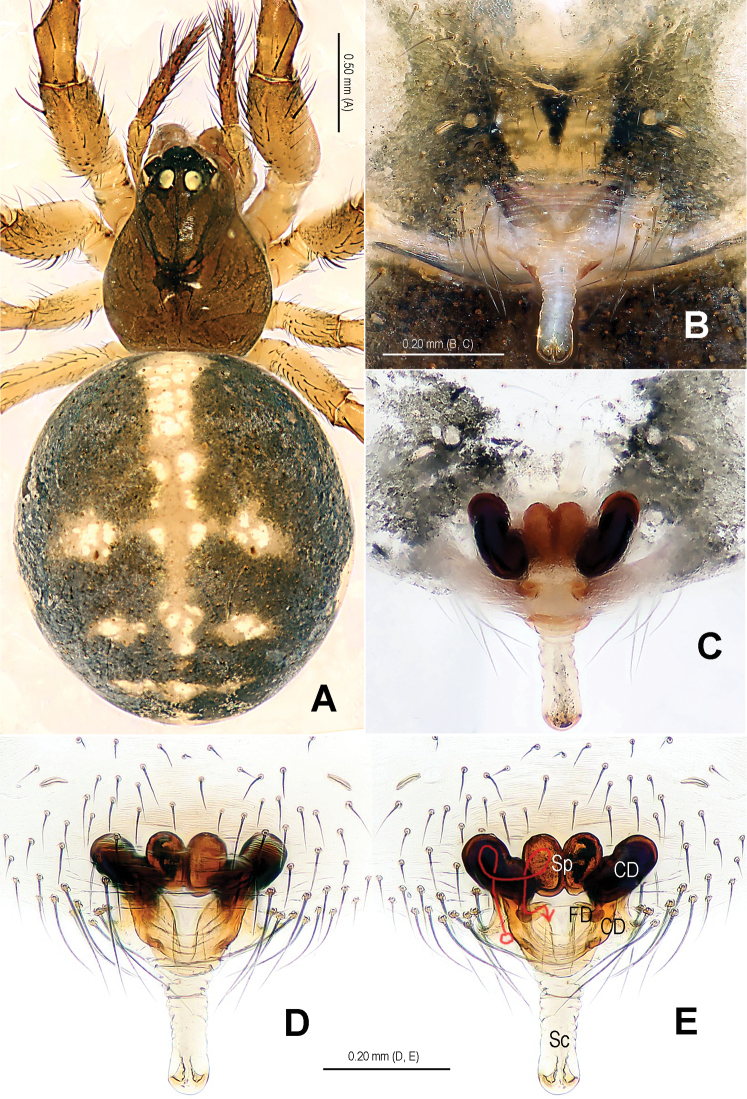
*Alaria bicornis* sp. n., female paratype, from Tham Kieo. **A** Habitus, dorsal **B** Epigyne, ventral **C** Vulva, dorsal **D** Epigyne (lactic acid-treated), ventral **E** Vulva, dorsal (red line showing course of duct system). CD = copulatory duct; FD = fertilization duct; Sc = scape; Sp = spermathecae.

**Figure 14. F14:**
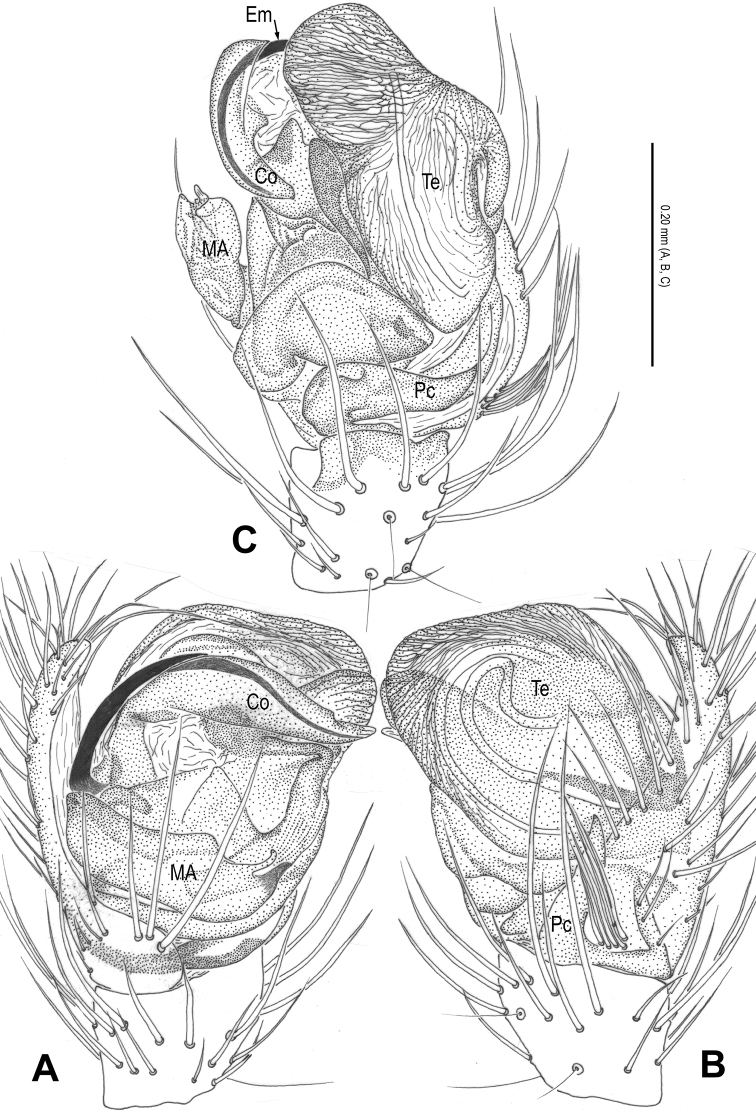
*Alaria bicornis* sp. n., holotype male. **A** Palp, prolateral **B** Ditto, retrolateral **C** Ditto, ventral. Co = conductor; Em = embolus; MA = median apophysis; Pc = paracymbium; Te = tegulum.

**Figure 15. F15:**
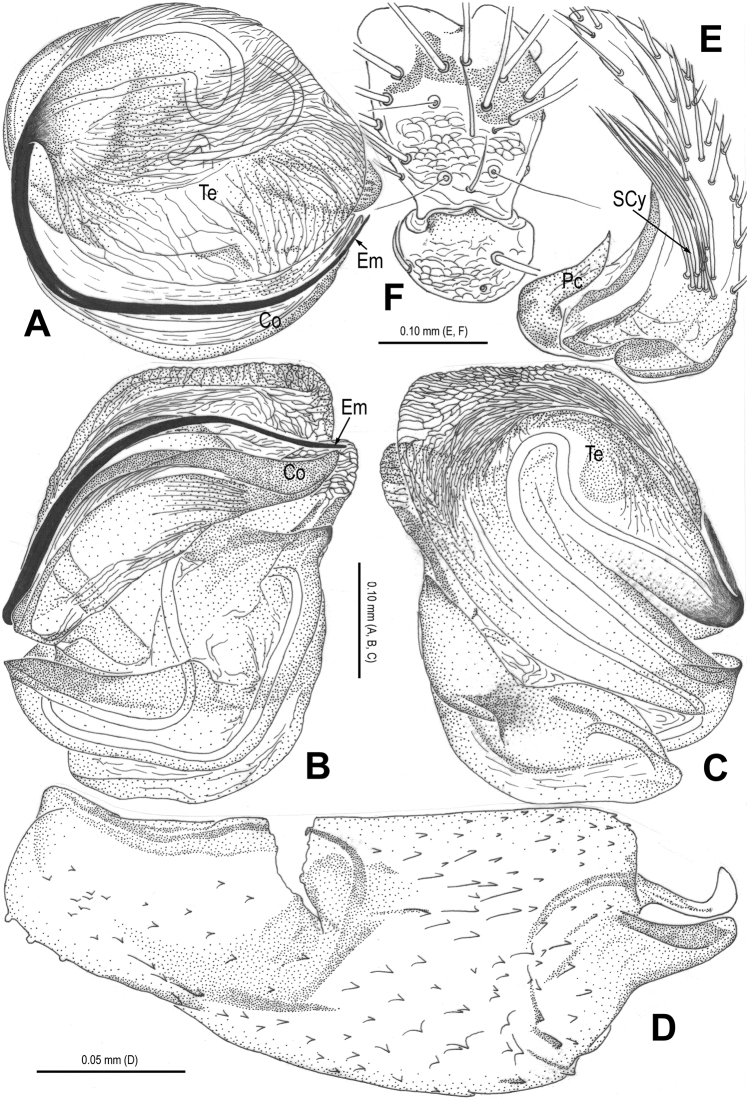
*Alaria bicornis* sp. n., holotype male. **A** Bulb (median apophysis removed), distal **B** Ditto, prolateral **C** Ditto, retrolateral **D** Median apophysis, prolateral **E** Cymbium, retrolateral **F** Palpal patella and tibia, ventral. Co = conductor; Em = embolus; Pc = paracymbium; SCy = setae of cymbium; Te = tegulum.

**Figure 16.**
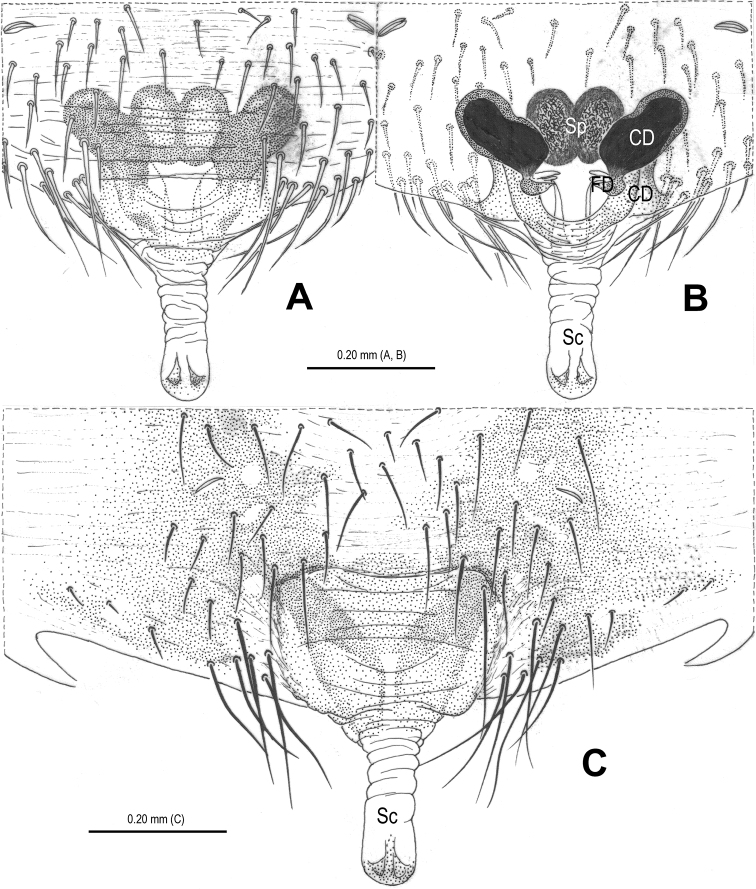
*Alaria bicornis* sp. n., female paratype, from Tham Kieo. **A** Epigyne (lactic acid-treated), ventral **B** Vulva (lactic acid-treated), dorsal **C** Epigyne (untreated), ventral. CD = copulatory duct; FD = fertilization duct; Sc = scape; Sp = spermathecae.

This new species and *Alaria chengguanensis* (Zhao and Li 2012: figs 1A–D, 2A–B, 3A–D, 4A–B, 5A–B) share the combination of the following features: tufted setae of cymbium (Figs 12E, 15E), especially large median apophysis (Figs 11B, 12D, 14A, 15D), and long embolus mostly enveloped by conductor in male, an utterly exposed scape protruding from beneath epigynal posterior margin and similar configurations of vulva in female, but the new species can be distinguished from the latter by the developed, strongly rugose tegulum (Figs 11C, 12C, 15C), the large median apophysis with 2 distal processes (Figs 12D, 14A, 15D) and the absence of a hooked process in paracymbium in male (Figs 12E, 15E), the oval median spermathecae (Figs 13C, E, 16B), the strongly sclerotized, long oval, lateral coils of copulatory duct (Figs 13E, 16B) and the narrow scape with two hoods in female (Figs 13C–E, 16A–C).

#### Description.

Habitus see in Figs 11A, 13A. DS pear-shaped, brownish yellow, thoracic fovea black, with symmetric dark veins. Eyes with black bases. Cervical groove distinct. Sternum brown. Legs proximally yellow, distally dark yellow. Opisthosoma oval, dark, dorsum with a long, longitudinal white stripe and 3 pairs of white spots minishing in sequence.

Male palp (Figs 11B–D, 14A–C): patella with 1 strong macroseta (Figs 12F, 15F). Tibia with 3 trichobothria (Figs 12F, 15F). Paracymbium horn-shaped, proximally large, distally pointed (Figs 12E, 15E). Tegulum broad, strongly rugose and sclerotized (Figs 12A, 12C, 15A, 15C). Median apophysis very large, surface with tiny aculei, with one fingerlike and one hooked process (Figs 12D, 15D). Conductor chisel-shaped, distinctly sclerotized, distally horn-shaped (Figs 11B, 11D, 12A, 14A, 14C, 15A). Embolus long, needle-shaped, sclerotized, most part embedded in conductor, embolic tip just on distal end of conductor (Figs 12A–B, 14A, 14C, 15A–B).

Female copulatory organ (Figs 13B–E, 16A–C): epigynal area covered with setae (Figs 13D, 16C). Scape long, fingerlike, rugose and membranous, distally weakly sclerotized, with 2 small hoods, extending from epigynal posterior margin (Figs 13B–C, 16A–C). Spermathecae oval, juxtaposed; lateral copulatory ducts strongly sclerotized, overlapping with dorsally posterolateral part of medial spermathecae (Figs 13C, 13E, 16B). Copulatory ducts wide, leading to posterolateral part of spermathecae (Figs 13E, 16B). Fertilization ducts short, arising posterolaterally from spermathecae (Figs 13E, 16B).

Male: Total length 2.01, DS 0.99 long, 0.78 wide, clypeus 0.15, sternum 0.60 long, 0.46 wide, coxae IV separated by their width, opisthosoma 1.09 long, 1.00 wide. PME separated by less than half their diameter. Macrosetae: Leg I: femur d2, p1, r1, patella d2, tibia d2, p2, r1, v2, metatarsus d1, r1, v1; leg II: femur d2, r1, patella d2, tibia d1, r1, v1, metatarsus d1, r1, v1; leg III: femur d2, patella d2, tibia p1, r1, v1, metatarsus d1, p1, r1; leg IV: femur d2, p1, patella d2, tibia d1, p1, r1, v1, metatarsus d1, p1, r1. Metatarsal trichobothria: Tm I: 0.24; Tm II: 0.24; Tm III: 0.09. Leg measurements: I 3.02 (0.97, 0.40, 0.66, 0.62, 0.37); II 2.44 (0.80, 0.35, 0.50, 0.46, 0.33); III 1.82 (0.56, 0.24, 0.34, 0.38, 0.30); IV 2.31 (0.73, 0.31, 0.48, 0.49, 0.30).

Female (collected together with holotype): total length 2.95, DS 1.10 long, 0.90 wide, clypeus 0.11, sternum 0.61 long, 0.47 wide, coxae IV separated by their width, opisthosoma 1.93 long, 1.75 wide. PME separated by about half their diameter. Macrosetae: Leg I: femur p1, r1, patella d2, tibia d2, p2, r1, v2, metatarsus p1, r2; leg II: femur d1, r1, patella d2, tibia d2, p1, r1, v1, metatarsus p1, r1, v1; leg III: femur d1, patella d2, tibia d1, r1, v1, metatarsus d1, p1, r1, v1; leg IV: patella d2, tibia d1, p1, r1, metatarsus p1, r1. Metatarsal trichobothria: Tm I: 0.26; Tm II: 0.23; Tm III: 0.12. Leg measurements: I 3.65 (1.20, 0.44, 0.80, 0.76, 0.45); II 2.99 (0.94, 0.40, 0.63, 0.60, 0.42); III 2.05 (0.61, 0.28, 0.39, 0.42, 0.35); IV 2.88 (0.90, 0.33, 0.65, 0.60, 0.40).

#### Variation.

The total length ranges from 1.88 to 2.15 in males (n = 5) and from 2.73 to 3.20 in females (n = 15).

#### Distribution.

See in Fig. 19.

### Genus Chthonopes

Wunderlich, 2011

http://species-id.net/wiki/Chthonopes

#### Type species.

*Chthonopes jaegeri* Wunderlich, 2011 from Laos.

The genus was described in 2011 from two species recorded in Laos (Wunderlich 2011). The type species was known from Bolikhamsay Province from its type locality, *Chthonopes cavernicolus* Wunderlich, 2011 was recorded from Khammouan Province. Two additional females from Oudomxai Province were preliminary assigned to *Chthonopes* as well without describing them formally as new species. All spiders were recorded from caves.

### Chthonopes thakekensis sp. n.

http://zoobank.org/92AA0D92-9434-414D-A86F-4596C0164644

http://species-id.net/wiki/Chthonopes_thakekensis

Figs 17 – 19

#### Material examined.

**Laos: *Khammouan Province*:** Holotype: ♀ (SMF), Thakek area, Ban Phôungam-Mai, 17°32.954'N, 104°48.754'E, altitude 180 m, Tham Phayat, in limestone cave, leg. 25 November 2012.

#### Etymology.

This specific name is taken from type locality; adjective.

#### Diagnosis.

**Figure 17.**
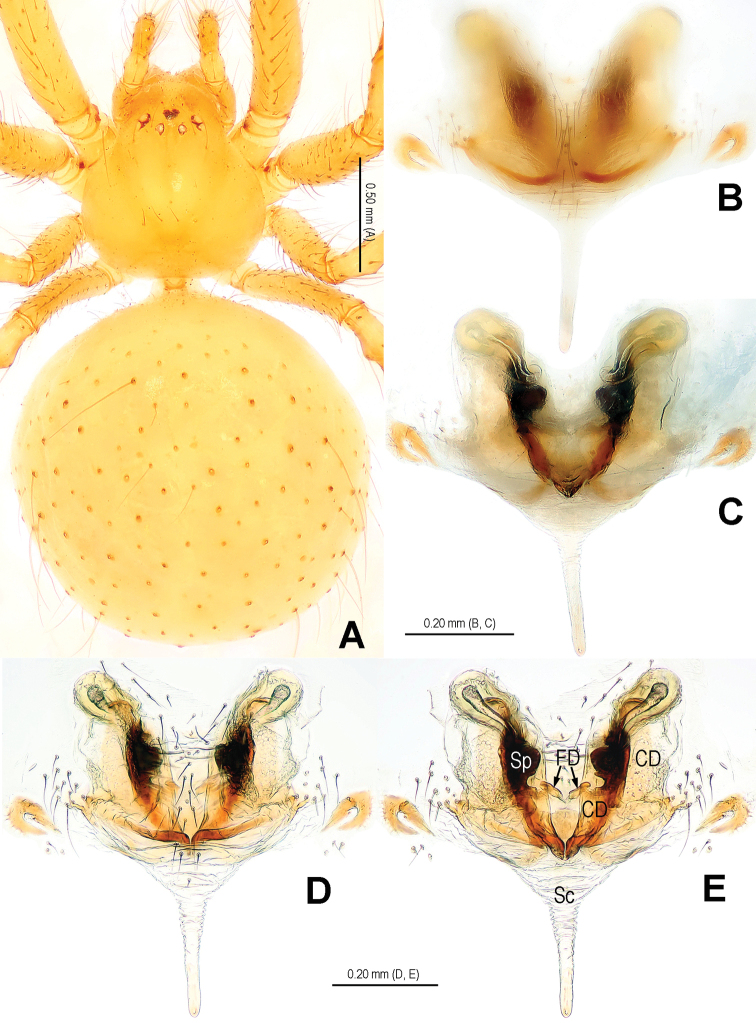
*Chthonopes thakekensis* sp. n., holotype female. **A** Habitus, dorsal **B** Epigyne (untreated), ventral **C** Vulva, dorsal **D** Epigyne (lactic acid-treated), ventral **E** Vulva, dorsal. CD = copulatory duct; FD = fertilization duct; Sc = scape; Sp = spermathecae.

**Figure 18.**
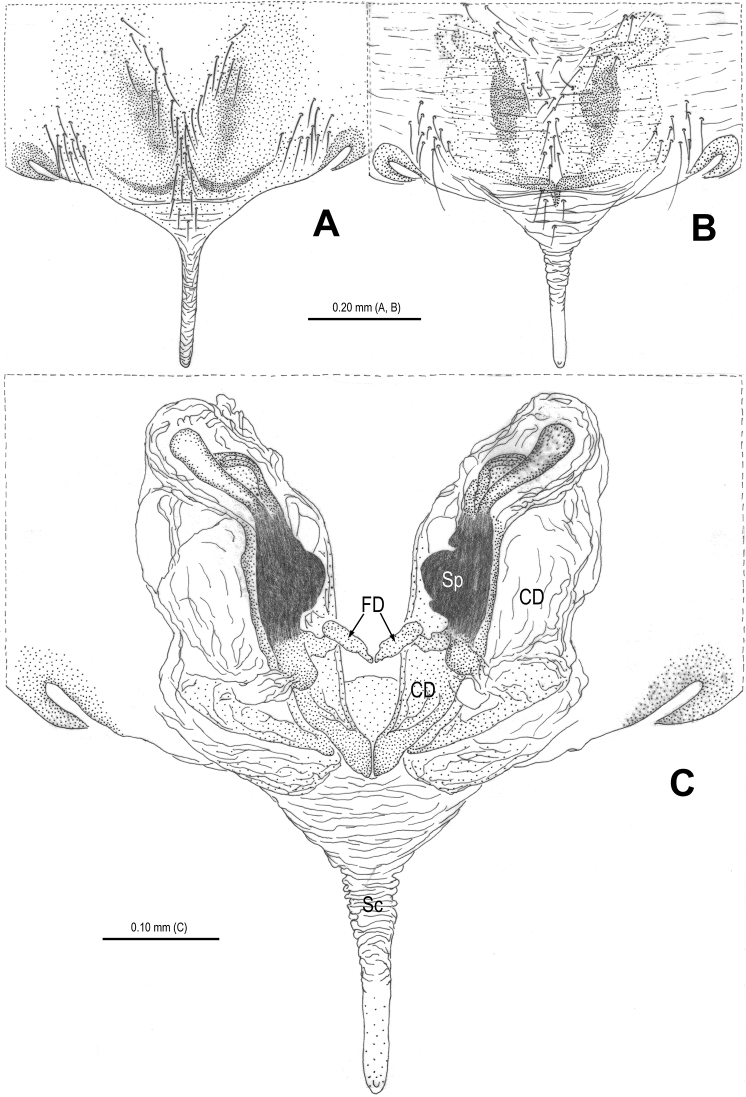
*Chthonopes thakekensis* sp. n., holotype female. **A** Epigyne (untreated), ventral **B** Ditto (lactic acid-treated), ventral **E** Vulva (lactic acid-treated), dorsal. CD = copulatory duct; FD = fertilization duct; Sc = scape; Sp = spermathecae.

The new species is similar to *Chthonopes jaegeri* Wunderlich, 2011 (see Wunderlich 2011: 443, figs 18d–f) in the shape of epigyne and the configurations of vulva, but can be distinguished from it by the presence of translucent accessory spermathecae (Figs 17D–E, 18C; absent in *Chthonopes jaegeri*) and the large, semi-circular main spermathecae (Figs 17E, 18C; circular in *Chthonopes jaegeri*).

#### Description.

Habitus see in Fig. 17A. DS yellow, pear-shaped, smooth; eyes small, with black base, AME contiguous, LE contiguous, anterior eye row recurved, posterior eye row procurved; sternum yellow, with sparse setae; legs yellow; opisthosoma spherical, covered with sparse long setae, setal base sclerotized.

Female copulatory organ (Figs 17C, 17E, 18C): epigyne large, with long setae in midline (Figs 17B, 18A); scape long, translucent, rugose, extending from posterior margin of epigynal plate, distal end weakly sclerotized (Fig. 17D); Spermathecae large, strongly sclerotized, separated by about 1.2 times their width (Fig. 17E); accessory spermathecae claviform, translucent (Fig. 17E); copulatory ducts wide, rugose, sclerotized, connected with posterior margin of main spermathecae (Figs 17E, 18C); fertilization ducts originating medio-posteriorly from main spermathecae, apical parts close to each other (Figs 17E, 18C).

Female: Total length 2.51, DS 0.89 long, 0.85 wide, clypeus 0.18, sternum 0.48 long, 0.49 wide, coxae IV separated by 0.97 time their width, opisthosoma 1.63 long, 1.60 wide. PME separated by about 1.5 times their diameter. Macrosetae: Leg I: patella d2, tibia d2, p2, r1, metatarsus d2, p5, r2, v2; leg II: patella d2, tibia d6, p3, r2, v3, metatarsus d2, p4, r2, v4; leg III: patella d2, tibia d2, p3, r3, v3, metatarsus d2, p6, r4, v6; leg IV: patella d2, tibia d3, p3, r4, metatarsus d3, p4, r3, v2. Metatarsal trichobothria: Tm I: 0.20; Tm II: 0.15; Tm III: 0.27. Leg measurements: I 3.83 (1.17, 0.49, 0.83, 0.79, 0.55); II 3.40 (1.03, 0.42, 0.74, 0.69, 0.52); III 2.56 (0.76, 0.34, 0.51, 0.54, 0.41); IV 3.03 (0.95, 0.36, 0.68, 0.62, 0.42).

Male unknown.

#### Distribution.

See in Fig. 19.

**Figure 19.**
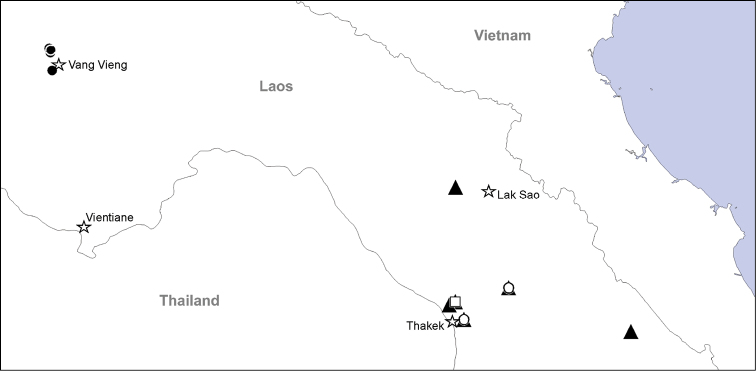
Records of four species of Theridiosomatidae from Laos. Filled triangles –*Alaria cavernicola* sp. n.; Open circles – *Alaria navicularis* sp. n.; Filled circles – *Alaria bicornis* sp. n.; Open square – *Chthonopes thakekensis* sp. n., Stars — towns.

## Supplementary Material

XML Treatment for Alaria

XML Treatment for Alaria cavernicola

XML Treatment for Alaria navicularis

XML Treatment for Alaria bicornis

XML Treatment for Chthonopes

XML Treatment for Chthonopes thakekensis
